# Steady state motion of a shear deformable beam in contact with a traveling surface

**DOI:** 10.1007/s00707-019-02476-x

**Published:** 2019-08-09

**Authors:** Evgenii Oborin, Yury Vetyukov

**Affiliations:** 1 Institute of Technical Mechanics, Johannes Kepler University Linz, Altenbergerstr. 69, 4040 Linz, Austria; 2 Institute of Mechanics and Mechatronics, Vienna University of Technology, Getreidemarkt 9, 1060 Vienna, Austria

## Abstract

A shear deformable beam moving along a straight path is considered as an idealization of the problem of stationary operation of a belt drive. The partial contact with a traveling surface results in the shear deformation of the beam. The tangential contact force grows near the end of the contact zone. Assuming perfect adhesion of the lower fiber of the beam to the traveling surface (no slip), we analytically demonstrate the necessity of accounting for concentrated contact forces and jump conditions, which is important for modeling the belt–pulley interaction. Along with dynamic effects, we further consider a frictional model with zones of stick and slip contact and demonstrate its convergence to the results with perfect adhesion at growing maximal friction force.

## Introduction

Belt drive mechanics is an extensively studied research area with applications in power transmissions, conveyors, elevators, and processing of metals and polymers. It is natural to investigate the belt drive mechanics with one-dimensional structural models of strings and rods because of the high length-to-thickness ratio; many publications are devoted to this topic, including the monograph [17]. In the contact problem for strings and rods, particular attention should be paid to the transition conditions at the boundaries between the contact and free zones, which we call “touching points” in the following. It is known that concentrated contact forces at these touching points result with Kirchhoff (shear undeformable) rod models even in static problems with frictionless contact; see [2, 5, 6, 11, 19, 32]. The perfect adhesion (no-slip) condition between the moving belt and the surface of a rotating pulley also results in concentrated tangential contact forces in the case of a string model of the belt [14, 30, 33]. It is, however, known that the singularity in the normal contact force vanishes with the introduction of shear flexibility of the rod, see contributions [3, 7], in which the tensioning of the belt on the pulleys is treated. It is one of the aims of the present study to answer the question whether shear flexibility eliminates the concentrated tangential contact forces for moving belts as well.

The alternative to the idealized model with perfect adhesion is the admission of Coulomb’s friction law and the corresponding analysis of the developing zones of sticking and sliding contact. This behavior is traditionally called “belt creep”; see, e.g., [27] for an exact analytical solution of the problem of steady state motion of an extensible belt with no bending stiffness. A whole body of publications is related to the transient finite element analysis featuring string and rod models for the belt as well as various simplifications of the friction law; see, e.g., [10, 12] for conventional finite element approaches. Putting the mixed Eulerian–Lagrangian kinematics into the basis of a non-material finite element formulation [28, 29, 31] allows avoiding frequent switching of contact states within single elements. This approach is getting increasingly popular for modeling belt drives and similar structures; see [18, 23, 25, 26]. The latter reference provides also an analytical solution for the transient growth of sliding zones during the operation of an idealized belt drive.

The present study is focused on the effect the transverse shear has on the nature of the belt–pulley contact interaction, which appears to be important owing to the following reasons.

- Shear flexibility essentially influences the distribution of the contact pressure between the belt and the pulleys; see [1, 3, 9, 16].
- Actual power transmission belts (V-belts) often feature thin steel fibers embedded in a rubber body. This reinforcement results in relatively high shear flexibility in comparison with extensional and bending ones. The belt creep is then mainly determined by shear deformation, which makes it quite different from the case of flat belts without reinforcement; see [15] for one of the first considerations of this effect.

Results of the present paper contribute to the broader research topic, which aims at efficient mathematical modeling of the steady state motion of a belt in contact with two rotating pulleys, including contact conditions and dynamic effects—in the spirit of the analysis presented in [22], but with no geometric simplifications. Smallness of the creep (sliding) zones in belt drives at realistic operation conditions makes it desirable to develop an idealized model with perfect adhesion (no-slip condition) on the entire contact surface similar to the previous analysis with the string model [14, 30, 33]. The attempt to extend the static equations to the case of a moving belt [3] and to solve the resulting boundary value problem [4] has not succeeded so far. Trying to solve the problem using various finite element schemes, we arrived at the conclusion that its formulation is probably incomplete without the consideration of concentrated contact forces in the touching points. Being known from the essentially simpler string model, this effect is far less expected to be crucial for shear deformable beams with their rich kinematics and thus deserves being studied under simplifying assumptions.

**Fig. 1:**
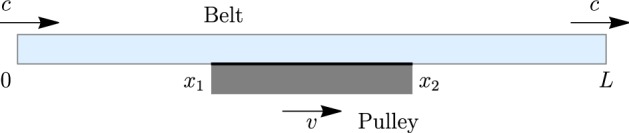
Belt drive as straight axially moving beam in frictional contact with pulley as rigid moving plate

In the following, we present a simple model of a moving belt drive, specially designed to investigate the contact interaction as depicted in Fig. 1.

A straight beam with extension and shear moves along the *x* axis according to the prescribed rate *c* of the material influx at the left end and outflux at the right end of the control domain $0\le x\le L$; see [29, 31] for an in-depth discussion of this sort of boundary conditions. The middle segment of the beam $x_1\le x\le x_2$ is supported by a rough surface moving with the velocity *v*. The small difference between the “desired” velocity of the beam *c* and the velocity *v*, which is imposed on the lower fiber of its middle part, results in the deformation of the beam and a complicated distribution of the contact interactions.

The below presented analytical solutions are based on the following simplifying assumptions.

- The transverse deflections of the beam are excluded from the consideration, i.e., the beam is assumed to be pressed against a horizontal surface, which is partially frictionless and partially rough and moving (the latter part we denote as “contact segment”).
- The small strain theory simplifies the analysis, allowing making no distinction between the derivatives with respect to the material and the spatial coordinates.
- Both the quasistatic and dynamic cases are considered under the assumption of steady state motion, when the observed deformations and velocities vary along the considered control domain, but remain unchanged in time at a given point *x*.
- The perfect adhesion (no-slip) condition between the lower fiber of the beam and the moving surface is imposed in the segment of contact.

As demonstrated in Sect. 2.3, the above statement of the problem inevitably leads to a concentrated contribution to the tangential contact force between the beam and the moving rough surface such that the entire contact interaction comprises a distributed part and the single force in the point $x_2$. The argumentation rests heavily on the condition of conservation of the total material length of the beam within the control domain, which follows from the considered type of boundary conditions and corresponds to the case of a closed belt. Releasing the last (perfect adhesion) assumption in Sect. 4, we also obtained solutions with zones of sticking and sliding friction in the contact segment, which required the involvement of numerical equation solving. Importantly, a series of numerical examples in Sect. 5 demonstrate that the latter solutions converge to the results of the no-slip model when the maximal friction force (assumed equal for sticking and sliding zones) is increased and the creep zone shrinks to a point.

## Quasistatic solution

### Problem formulation

A straight extensible and shear deformable beam with thickness *h*, bending stiffness *a*, tension stiffness $b_1$, and shear stiffness $b_2$ is pressed against a straight surface such that transverse deflections are not possible. The scheme is depicted in Fig. 1. At the points $x=0$ and $x=L$, the axial motion of the beam is kinematically imposed: Material length *c* enters the domain per unit time from the left and leaves the domain at the right. The material length of the beam inside the control domain is thus kept constant and is assumed to be equal *L*. As small deformations are considered, this corresponds to the condition that the integral of the linearized strain over the entire domain vanishes,

1 $$\begin{aligned} \int _0^L\varepsilon (x)\,\mathrm {d} x = 0. \end{aligned}$$

In this section, we also restrict the consideration to small velocities, such that the inertia terms are neglected, which is the assumption of quasistatics.

The control domain is divided into three segments: two free segments $0<x<x_1$ and $x_2<x<L$ and a contact segment $x_1\le x\le x_2$ in between. The beam is sliding with no friction in the left and in the right segments and sticks to a traveling surface in the middle one. The velocity of the surface *v* is slightly different from *c*. Just the lower fibers of the beam move together with the surface, which means that the middle line of the beam may still deform. Consider the steady state regime of motion, in which the velocities of particles, the strains, the forces, and the moments observed at a given point $x=\mathrm {const}.$ do not change in time; this means that the strain in the lower fiber in the sticking segment is

2 $$\begin{aligned} \varepsilon _{\mathrm {bottom}} = v / c - 1. \end{aligned}$$

This relation between the material velocity *v* and the strain in a steady state process follows with the introduction of a material coordinate in the beam; for details, we refer to [14]; see also [24, 27]. The choice of the strain measure (Biot, Green–Lagrange, etc.) is irrelevant in the present context of small strain analysis.

Along with the axial strain of the middle line, the kinematics of the string is described by the rotation angle of the cross sections $\theta (x)$. As there are no transverse deflections, the shear strain equals to $-\theta$, and we proceed with the linear constitutive relations:

3 $$\begin{aligned} \begin{aligned}&M = a \theta ^{\prime },\\&Q = -b_2 \theta , \\&T = b_1 \varepsilon , \end{aligned} \end{aligned}$$

where *M* is the moment in the belt, *Q* is the shear force, and *T* is its tension. Three stiffness coefficients have been introduced: *a* is the bending stiffness, $b_1$ is the tension stiffness, and $b_2$ is the shear stiffness. A prime denotes the differentiation with respect to the spatial axial coordinate *x* (which is equal to the differentiation with respect to the material coordinate under the assumption of small strains).

The following balance equations contain the tangential contact force *f*(*x*), which is assumed acting on the lower surface of the beam (thus resulting in moment loading *f*(*x*)*h* / 2 too) and may be nonzero only in the contact segment:

4 $$\begin{aligned} \begin{aligned}&T^{\prime } + f = 0, \\&M^{\prime } + Q + \frac{h}{2}f =0. \end{aligned} \end{aligned}$$

### Segment of sticking contact

In the segment of sticking contact, the beam bottom has a constant tension strain. The strain of the fiber on the bottom of the beam follows from the Timoshenko beam kinematics as

5 $$\begin{aligned} \varepsilon _{\mathrm {bottom}} = \varepsilon + \frac{h}{2}\theta ^{\prime }. \end{aligned}$$

A single differential equation for the angle $\theta$ follows after eliminating the distributed force *q* and demanding that $\varepsilon '_{\mathrm {bottom}} = 0$,

6 $$\begin{aligned} \begin{aligned}&\theta ^{\prime \prime }-\alpha ^2\theta = 0, \\&\alpha \equiv 2\sqrt{\frac{b_2}{4a+b_1h^2}}. \end{aligned} \end{aligned}$$

We solve this equation and find $\theta$, $\varepsilon$, and *q* in the segment of sticking contact up to the accuracy of two integration constants $C_{1,2}$:

7 $$\begin{aligned} \begin{aligned}&\theta _\mathrm {stick} = \exp {\left( \alpha x\right) } C_1 + \exp {\left( -\alpha x\right) } C_2, \\&\varepsilon _\mathrm {stick} = \frac{v}{c} - 1 - \frac{h\alpha }{2}\left[ \exp {\left( \alpha x\right) }C_1 - \exp {\left( -\alpha x\right) } C_2\right] ,\\&f_\mathrm {stick} = \frac{b_1 h \alpha ^2}{2} \left[ \exp {\left( \alpha x\right) }C_1 - \exp {\left( -\alpha x\right) } C_2\right] . \end{aligned} \end{aligned}$$

### Discussion of continuity

Following the previously conducted static analysis of the geometrically nonlinear model [3], we first try assuming that there are no concentrated contact interactions in the end points of the segment of sticking (touching points) $x=x_{1,2}$, such that the force and the moment are continuous in the entire domain $0< x < L$. This implies the continuity of $\varepsilon$ and $\theta ^{\prime }$, and for the segments to the left and to the right of the sticking one, we find that

8 $$\begin{aligned} \begin{aligned}&\left. \varepsilon \right| _{x<x_1} = \varepsilon _{\mathrm {left}} = \varepsilon _{\mathrm {stick}}(x_1), \\&\left. \varepsilon \right| _{x>x_2} = \varepsilon _{\mathrm {right}} = \varepsilon _{\mathrm {stick}}(x_2), \end{aligned} \end{aligned}$$

and the two functions $\left. \theta \right| _{x<x_1}$ and $\left. \theta \right| _{x>x_2}$ are determined by the balance of moments (Eq. (4)), with $f=0$, and contain two integration constants each. Altogether this results in six integration constants in $\theta$ in three segments.

We adopt the boundary conditions that $\theta =0$ at $x=0,L$ (we could also have considered an infinite domain with vanishing $\theta ^{\prime }$ at the infinity, which does not make the situation different as the number of conditions remains the same), we also have matching conditions for $\theta$ and $\theta ^{\prime }$ at $x=x_{1,2}$. The continuity of $\theta$ is the kinematic condition which cannot be violated in the continuous beam. The continuity of $\theta ^{\prime }$ (and of the bending moment *M*) at the touching points is assumed in the present context and will be examined by the appropriate results. Boundary and continuity conditions pose six constraints, which fully determine all the constants and the solution. But there is one more condition of the conservation of the material length, which was not imposed so far: total integral of the strain $\varepsilon$ over the control domain must vanish, see Eq. (1), which cannot generally be satisfied with the currently approved assumptions. Three options are thinkable.

1. We release the condition of the conservation of the material length of the control domain and find a unique continuous solution. This may be of theoretical interest, but is not relevant for the present purpose of solving a model problem for the more general case of a moving belt: the belt is closed, its material length is preserved, and experiments with finite element solutions of the belt problem clearly indicate that the concentrated contact interaction needs to be included into the model to obtain reasonable solutions.
2. We admit a concentrated contact interaction in a single point. Condition of adhesion implies that the strain $\varepsilon _{\mathrm {bottom}}$ is continuous at the point $x_1$, in which material particles come into contact. Therefore, we now consider that
  9 $$\begin{aligned} f(x) = f_0(x) + F\delta (x-x_2), \end{aligned}$$
  in which $f_0$ is the continuous distribution determined above in Eq. (7), $\delta$ is the Dirac impulse function, and the concentrated force *F* (which also results in a concentrated moment) defines jumps in $\varepsilon$ and $\theta ^{\prime }$ in the right touching point. This allows satisfying the condition of constant material length, which is similar to the string solution introduced in [14]. Whether the concentrated force will be necessary if not a beam, but rather a 2D continuum is moving with the considered kinematic conditions, remains an open question.
3. We seek a continuous solution in the presence of slip (creep zone in the terminology of the theory of belt drives). The segment $x_1 \le x \le x_2$ is divided into a zone of sticking contact $x_1 \le x \le x_*$, in which the contact force does not exceed some maximal value, $f < f_*$, and a zone of sliding $x_*< x \le x_2$, in which we apply $f=f_*$ and the corresponding distributed moment. The sliding friction force $f_*$ is prescribed (we can argue that the beam is pressed against the surface so strongly that the normal contact force is almost constant, and use the conventional Coulomb’s friction law), the coordinate $x_*$ follows from the matching conditions.

The subsequent discussion in Sect. 2.4 rests upon the second of the mentioned options, and we remain with the assumption of quasistatics.

### Free segments and boundary conditions in quasistatic case

In the case with the concentrated contact force at the point $x=x_2$, we still use the matching conditions at $x=x_1$. The continuity of $\theta$ follows from the beam kinematics. No jumps in the force *T* and in the moment *M* are expected here because of the adhesion, which implies the continuity of the strain of the lower fiber $\varepsilon _\mathrm {bottom}$ at the point, where material particles of the beam come into contact with the traveling surface. We refer the interested reader to [14, 25, 33] for details. Using these conditions, we derive the solution in the left segment $0< x < x_1$:

10 $$\begin{aligned} \begin{aligned}&\varepsilon _{\mathrm {left}}=\left. \varepsilon _{\mathrm {stick}} \right| _{x=x_1}=\frac{v}{c}-1-\frac{h\alpha }{2}\left[ \exp {\left( \alpha x_1 \right) }C_1 - \exp {\left( -\alpha x_1 \right) } C_2\right] ,\\&\theta _{\mathrm {left}}=\exp {\left( -\alpha x_1 \right) }\left[ \exp {\left( \alpha 2x_1 \right) }C_1 + C_2\right] \cosh {(\beta x_1 )} \sinh {(\beta x)}, \end{aligned} \end{aligned}$$

where $\beta \equiv \sqrt{b_2/a}$. The solution in the left segment is now fully determined by the already introduced constants $C_{1,2}$. One of the conditions for finding these constants is the continuity of $\theta ^{\prime }$ (moment) between the segments:

11 $$\begin{aligned} x_1:\quad \theta '_{\mathrm {left}} = \theta '_{\mathrm {stick}}. \end{aligned}$$

In the present quasistatic problem, the jumps in the axial force *T* and in the bending moment *M* at the point $x_2$ are determined by the concentrated tangential contact force *F* and its moment relative to the middle line (there may also happen a jump in the transverse force *Q* because of a particularity in the normal contact force distribution, which is, however, not very important in the considered problem without deflection):

12 $$\begin{aligned} \begin{aligned}&[\![T ]\!]_{x_2} + F = 0,\\&[\![M ]\!]_{x_2} + \frac{h}{2}F = 0; \end{aligned} \end{aligned}$$

see [14] for consideration of jump condition in the belt drive mechanics and [20] for the general treatment of the jump conditions.

The first condition leads to the following expression for the tension strain in the right free segment $x_2< x< L$:

13 $$\begin{aligned} \varepsilon _{\mathrm {right}} = \frac{v}{c} - 1 - \frac{F}{b_1} - \frac{h\alpha }{2} \left[ \exp {\left( \alpha x_2 \right) }C_1 - \exp {\left( -\alpha x_2 \right) } C_2\right] , \end{aligned}$$

whereas the angle is derived from the kinematic matching condition $\left. \theta _{\mathrm {right}}\right| _{x=x_2} = \left. \theta _{\mathrm {stick}}\right| _{x=x_2}$:

14 $$\begin{aligned} \theta _{\mathrm {right}} = \exp {\left( -\alpha x_2 \right) }\left[ \exp {\left( \alpha 2x_2 \right) }C_1 + C_2\right] \cosh {(\beta (L-x_2) )} \sinh {(\beta (L-x))}. \end{aligned}$$

Two unknown constants $C_{1,2}$ follow from the two linear algebraic equations, namely from the matching condition for $\theta ^{\prime }$ at $x_1$ (Eq. 11) and from the second jump condition at $x_2$ (Eq. (12)). The explicit formulas for the constants are rather lengthy and are not provided in the present paper.

Finally, the concentrated force *F* follows from the condition of conservation of the material length (Eq. (1)):

15 $$\begin{aligned} x_1 \varepsilon _{\mathrm {left}} + (L-x_2) \varepsilon _{\mathrm {right}} + \int \limits _{x_1}^{x_2}{\varepsilon _\mathrm {stick}\mathrm {d} x} = 0. \end{aligned}$$

Results of computation for a particular set of parameters will be given in Sect. 5 in comparison with the results of the generalized cases.

## Dynamic solution

In the dynamic case, the linear and rotary inertia terms arise in the balance relations (Eq. (4)). Accounting for them requires the knowledge of the linear and angular accelerations of material particles. Denoting material time derivatives (i.e., derivatives with respect to the time *t* in a given material particles) by a dot, we establish a relation to the local time derivative, which is computed in a fixed spatial position *x*. Considering an arbitrary time-varying field $\varphi (x,t)$, we derive [23, 29]

16 $$\begin{aligned} {\dot{\varphi }}=\partial _t\varphi +\dot{x}\varphi ^{\prime }. \end{aligned}$$

The local time derivative $\partial _t\varphi$ vanishes for the considered steady state process, in which strains, velocities, etc. do not vary in time in a given point in space, and only the second convective term with the velocity of the particle $\dot{x}$ remains. Similar to Eq. (2), we find $\dot{x}=c(1+\varepsilon )$ and finally obtain

17 $$\begin{aligned} {\dot{\varphi }}=c(1+\varepsilon )\varphi ^{\prime }. \end{aligned}$$

Now we consider $\varphi = \dot{x}$ and find the linear acceleration of a particle:

18 $$\begin{aligned} {\ddot{x}} = c(1+\varepsilon )(c(1+\varepsilon ))^{\prime }=c^2(1+\varepsilon )\varepsilon ^{\prime }, \end{aligned}$$

which in the considered small strain case shall be linearized to ${\ddot{x}} = c^2\varepsilon ^{\prime }$. Further we set $\varphi =\theta$, apply formula (17) twice and find the linearized angular acceleration $\ddot{\theta }= c^2\theta ^{\prime \prime }$. With $\rho$ being the mass of the beam per unit length and *I* the distributed inertia moment, we rewrite the balance equations (4) with the inertia terms:

19 $$\begin{aligned} \begin{aligned}&T^{\prime } + f - \rho c^2 \varepsilon ^{\prime }= 0, \\&M^{\prime } + Q + \frac{h}{2}f - Ic^2 \theta ^{\prime \prime } = 0. \end{aligned} \end{aligned}$$

The constitutive equations (3) remain unchanged.

### Segment of sticking contact in dynamic case

In the segment of sticking contact, we again use the stick condition for the strain of the lower fiber $\varepsilon _\mathrm {bottom}$; see Eq. (3). Now the balance relations (19) lead to the following second-order differential equation for $\theta$:

20 $$\begin{aligned} \begin{aligned}&\theta ^{\prime \prime } - \alpha _\mathrm {dyn}^2 \theta = 0,\\&\alpha _\mathrm {dyn} \equiv 2 \sqrt{\frac{b_2}{4a-4Ic^2+h^2(b_1-\rho c^2)}}. \end{aligned} \end{aligned}$$

The exact solution to this second-order differential solution contains two yet unknown integration constants,

21 $$\begin{aligned} \begin{aligned}&\theta _\mathrm {stick} = \exp {(\alpha _\mathrm {dyn} x )} C_1 + \exp {(-\alpha _\mathrm {dyn} x )} C_2, \\&\varepsilon _\mathrm {stick} = \frac{v}{c} - 1 - \frac{h \alpha _\mathrm {dyn}}{2}\left[ \exp {(\alpha _\mathrm {dyn} x )}C_1 - \exp {(-\alpha _\mathrm {dyn} x)} C_2\right] , \\&f_\mathrm {stick} = \frac{h\left( b_1-\rho c^2\right) \alpha ^2_\mathrm {dyn}}{2} \left[ \exp {(\alpha _\mathrm {dyn} x )}C_1 + \exp {(-\alpha _\mathrm {dyn} x )} C_2\right] . \end{aligned} \end{aligned}$$

To derive the general solution for the entire belt, we need to consider:

1. the differential equations in the free segments $0< x < x_1$ and $x_2< x < L$,
2. the boundary conditions at the entry and exit points of the contact domain $x=0,L$,
3. the continuity (matching) and jump conditions between the adjacent segments in the touching points $x=x_{1,2}$, and
4. the conservation of the material length of the beam.

### Free segments and boundary conditions in the dynamic case

Similar to the quasistatic case, the matching conditions at $x_1$ for $\varepsilon$ and $\theta$ and the boundary condition at $x=0$ lead to the solution in the left free segment $0< x < x_1$:

22 $$\begin{aligned} \begin{aligned}&\varepsilon _\mathrm {left} = \frac{v}{c} - 1 - \frac{h \alpha _\mathrm {dyn}}{2}\left[ \exp {(\alpha _\mathrm {dyn} x_1)}C_1 - \exp {(-\alpha _\mathrm {dyn} x_1 )} C_2\right] ,\\&\theta _\mathrm {left} = \frac{1}{2} \exp {(-\alpha _\mathrm {dyn} x_1 + \beta _\mathrm {dyn}(x_1-x))} (\exp {(2\beta _\mathrm {dyn}x )}-1 ) \cdot \\&\qquad \quad \cdot \left[ \exp {( 2 \alpha _\mathrm {dyn} x_1)} C_1 + C_2 \right] (\coth {(\beta _\mathrm {dyn} x_1)} - 1), \\&\beta _\mathrm {dyn} \equiv \sqrt{\frac{b_2}{a-I c^2}}. \end{aligned} \end{aligned}$$

The constant $\beta _\mathrm {dyn}$ has been introduced to shorten the equations. In the following, we additionally demand the continuity in $\theta ^{\prime }$ (moment) between the left and stick segments (Eq. (11)).

The jumps in the axial force *T* and in the bending moment *M* (and, respectively, in the axial strain $\varepsilon$ and in the bending strain $\theta ^{\prime }$) at the point $x_2$ are again determined by the concentrated contact force *F* and its moment relative to the middle line,

23 $$\begin{aligned} \begin{aligned}&[\![T-\rho c^2 \varepsilon ]\!]_{x_2} + F = 0,\\&[\![M - Ic^2 \theta ^{\prime } ]\!]_{x_2} + \frac{h}{2}F =0. \end{aligned} \end{aligned}$$

While for the general treatment of jump conditions in dynamics using the laws of balance we refer to [20], here we simply point out that equations (23) represent a direct consequence of the differential equations (19). Indeed, considering *T*, *M*, $\theta ^{\prime }$, and $\varepsilon$ as functions with discontinuities at $x=x_2$, we obtain Dirac impulses $\delta (x-x_2)$ in their derivatives. Further balancing them and accounting for Eq. (9), we arrive at Eq. (23).

The first jump condition in Eq. (23) and the matching condition for the angle $\theta$ at $x_2$ allow writing the expressions for the axial strain and the angle in the right free segment $x_2< x < L$:

24 $$\begin{aligned} \begin{aligned}&\varepsilon _\mathrm {right} = \frac{v}{c} - 1 - \frac{F}{b_1 - \rho c^2} - \frac{h \alpha _\mathrm {dyn}}{2} \left[ \exp {\left( \alpha _\mathrm {dyn} x_2 \right) }C_1 - \exp {\left( -\alpha _\mathrm {dyn} x_2 \right) } C_2\right] , \\&\theta _\mathrm {right} = \exp {\left( -\alpha _\mathrm {dyn} x_2 \right) } \left[ \exp {\left( 2 \alpha _\mathrm {dyn} x_2 \right) }C_1 + C_2\right] \cosh {(\beta _\mathrm {dyn}(L-x_2))} \sinh {(\beta _\mathrm {dyn}(L-x))}. \end{aligned} \end{aligned}$$

The second relation in Eq. (23) is used together with the matching condition in Eq. (11) to determine constants $C_{1,2}$. (It is a system of two linear algebraic equations.)

As above, we need the condition of conservation of the material length to obtain the value of concentrated force *F*; condition Eq. (15) is valid here as well. The numerical results for a benchmark example will be given in Sect. 5.

## Presence of slip

Effects of small sliding at axial motion, which are frequently denoted as elastic microslip [21], were studied in detail for small axial deflections of a beam in contact with a rough surface in [8, 13]. The present analysis is different in the sense that not a single static configuration, but a regime of steady state motion of the beam needs to be determined, which essentially changes the formulation of the problem.

### Equations and boundary conditions

Here, we seek a continuous solution in the presence of slip (creep zone in the terminology of the theory of belt drives). The contact segment $x_1 \le x \le x_2$ is now divided into zone of sticking contact $x_1 \le x \le x_*$, in which the contact force does not exceed some maximal value, $f \le f_*$, and a zone of sliding $x_*< x \le x_2$, in which we apply constant traction $f=f_*$ and corresponding distributed moment $f_*h / 2$. The sliding friction force $f_*$ is prescribed. This corresponds to the assumptions of the perfect Coulomb’s friction law with constant normal pressure between the beam and the supporting surface, which determines the particular value of $f_*$. The coordinate $x_*$ is found from the matching conditions between the zones.

In the stick region $x_1 \le x \le x_*$, the general solution is the same as in Sect. 3.1 (Eq. (21)); it depends on two yet unknown constants, which are of course different from the above cases.

In the slip segment $x_*< x \le x_2$, we have $f(x)=f_*= \mathrm {const}$ and $m(x)=f_*h /2 = \mathrm {const}$. The balance relations take the form

25 $$\begin{aligned} \begin{aligned}&T^{\prime } + f_*- \rho c^2 \varepsilon ^{\prime } = 0, \\&M^{\prime } + Q + \frac{h}{2}f_*- Ic^2 \theta ^{\prime \prime } = 0. \end{aligned} \end{aligned}$$

After substituting constitutive relations (3), we get two uncoupled linear differential equations for $\varepsilon$ (first order) and $\theta$ (second order):

26 $$\begin{aligned} \begin{aligned}&\varepsilon ^{\prime } + f_*\gamma =0, \\&\theta ^{\prime \prime }-\beta _\mathrm {dyn} \theta + \frac{h f_*\beta _\mathrm {dyn}}{2b_2}=0,\\&\gamma \equiv \frac{1}{b_1 - \rho c^2}, \end{aligned} \end{aligned}$$

in which the combination of the prescribed values is denoted $\gamma$ for brevity.

The general solution to these equations contains three yet unknown integration constants:

27 $$\begin{aligned} \begin{aligned}&\varepsilon _\mathrm {slip} = -\gamma f_*x + G_1, \\&\theta _\mathrm {slip} = \frac{h f_*}{2 b_2} + \exp {(\beta _\mathrm {dyn} x)}H_1 +\exp {(-\beta _\mathrm {dyn} x)}H_2. \end{aligned} \end{aligned}$$

To derive the general solution for the whole belt, we also need to consider the following:

1. the differential equations in the free segments,
2. the boundary conditions at the end points of the domain $x=0,L$,
3. the continuity (matching) conditions between the adjacent segments in the touching points $x=x_{1,2}$, and
4. the conservation of the material length of the beam.

### Free segments and boundary conditions in case with slip

At the transition between the stick segment and the left free segment, we again have the continuity conditions for $\varepsilon$, $\theta$, and $\theta ^{\prime }$. Along with the boundary condition at $x=0$ they result in the same expressions for $\varepsilon$ and $\theta$ in the free left segment as above in Sect. 3.2 (Eq. (22)).

Using the continuity at the right touching point $x=x_2$ and demanding that $\theta =0$ at $x=L$, we derive the explicit formulas for $\varepsilon$ and $\theta$ in the right free segment:

28 $$\begin{aligned} \begin{aligned} \varepsilon _\mathrm {right}&= -\gamma f_*x_2 + G_1, \\ \theta _\mathrm {right}&= \frac{1}{2b_2}\left( h f_*+ 2 b_2 \exp {(-\beta _\mathrm {dyn} x_2)} \left[ \exp {(2\beta _\mathrm {dyn} x_2)} H_1 + H_2 \right] \right) \cdot \\&\quad \cdot \cosh {(\beta _\mathrm {dyn} (L- x_2))} \sinh {(\beta _\mathrm {dyn} (L- x))}. \end{aligned} \end{aligned}$$

In the (yet unknown) transition point $x=x_*$, we shall demand continuity of the axial stain $\varepsilon$, angle $\theta$, and its derivative $\theta ^{\prime }$ (moment).

Finally, we write the condition of the conservation of material length in the form:

29 $$\begin{aligned} x_1 \varepsilon _\mathrm {left} + (L - x_2) \varepsilon _\mathrm {right} + \int \limits ^{x_*}_{x_1}{\varepsilon _\mathrm {stick}\mathrm {d}x} + \int \limits ^{x_2}_{x_*}{\varepsilon _\mathrm {slip}\mathrm {d}x}. \end{aligned}$$

The three continuity conditions at $x_*$ together with the continuity of $\theta ^{\prime }$ at $x=x_{1,2}$ and the constancy of material length (Eq. (29)) compose a nonlinear system of equations for the following six unknowns: two constants $C_{1,2}$ of the general solution in the stick segment, three constants $G_1$, $H_{1,2}$ of the solution in the slip segment, and the boundary coordinate $x_*$ between the segments. This system may be solved numerically by means of the standard Newton methods implemented in computer algebra systems (for instance, with function FindRoot in Wolfram Mathematica^1^).

## Numerical results

In this section, we present the numerical results for a benchmark example. Three considered models (quasistatic with perfect adhesion, dynamic with perfect adhesion, and dynamic with the zone of slip) shall be compared such that the influence of the specific effects is made visible.

The model parameters are: $L=1\,\mathrm {m}$ is the length of the considered part of the belt, coordinates $x_1 = 1/3\,\mathrm {m}$ and $x_2=2/3\,\mathrm {m}$ determine the location and length of the traveling contact surface (pulley), $h=0.1\,\mathrm {m}$ and $w=0.1\,\mathrm {m}$ are the height and the width of the cross section of the belt, $E=5 \cdot 10^7\,\mathrm {Pa}$ is the Young’s modulus, $\nu =0.45$ is the Poisson coefficient, and $\rho _3=1500\,\mathrm {kg/m^3}$ is the volume material density of the belt.

The elasticity of the belt is characterized by the three stiffness factors: $a=Ewh^3/12$ is the bending stiffness, $b_1=Ewh$ is the tension stiffness, and $b_2=Ekwh/2(1+\nu )$ is the shear stiffness with the shear correction factor $k=1.1$. The inertia of the belt is prescribed by $I=\rho _3 h w(h^2 + w^2)/12$, which is the rotary inertia moment, and by $\rho = \rho _3 h w$, which is the mass per unit length.

Before the considered domain ($x<0$) and after it ($x>L$), the velocity of the belt is prescribed to have the value $c= 120\,\mathrm {m/s}$. This high velocity of the belt results in a noticeable effect of inertia, as the dynamic contribution $\rho c^2=2.16\cdot 10^5\,\mathrm {N}$ in the denominator of the parameter $\gamma$ (see Eq. (26)) becomes comparable with the tension stiffness $b_1=5\cdot 10^5\,\mathrm {N}$. The rough contact surface (pulley) is moving with the velocity $v=121.2\,\mathrm {m/s}$, which results in the positive extension $\varepsilon$ and positive contact forces *f*. For the case with slip, we use the prescribed value of the maximal friction force $f_*=10^5\,\mathrm {N/m}$.

**Fig. 2:**
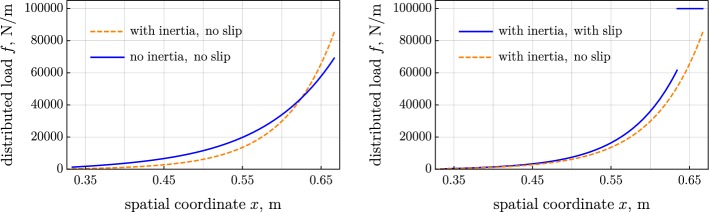
Distributed friction force over spatial coordinate: (left) inertia versus no inertia and (right) slip versus no slip

**Fig. 3:**
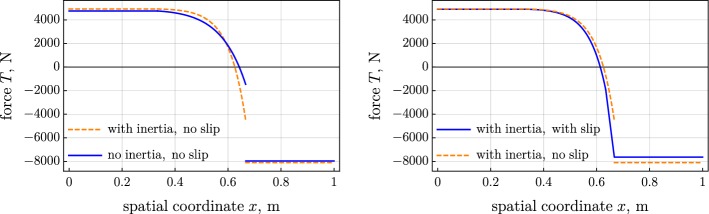
Tension over spatial coordinate: (left) inertia versus no inertia and (right) slip versus no slip

**Fig. 4:**
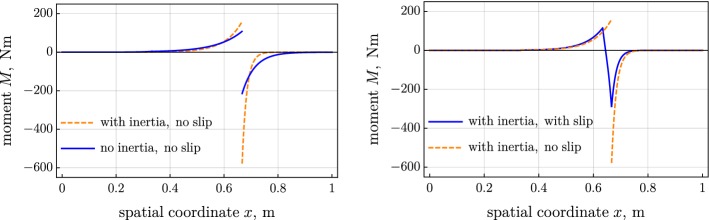
Moment over spatial coordinate: (left) inertia versus no inertia and (right) slip versus no slip

Figure 2 shows the distribution of the contact force, computed for the three considered variants of the model. Figures 3 and 4 present the tension and the moment in the belt. The introduced inertia makes the friction force more localized, which results in the steeper tension and moment; see left parts of Figs. 2, 3, and 4. The jump in the moment is noticeably larger in the inertial case than the jump in the quasistatic case; see left part of Fig. 4. On the contrary, the jump in the tension is considerably smaller in the inertial case than in the quasistatic one. The slip increases the distributed friction force and smoothens the jump in the tension and moment; see the right parts of Figs. 2, 3, and 4.

**Fig. 5:**
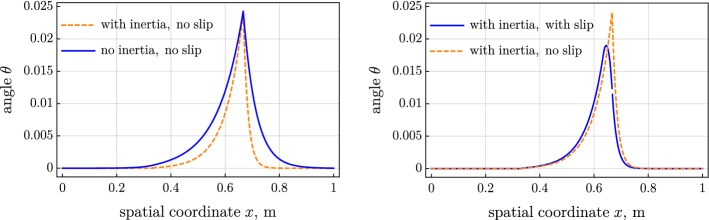
Angle over spatial coordinate: (left) inertia versus no inertia and (right) slip versus no slip

**Fig. 6:**
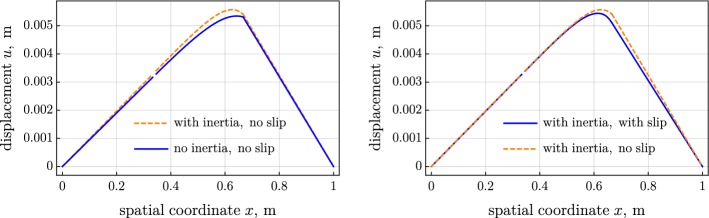
Displacement over spatial coordinate: (left) inertia versus no inertia and (right) slip versus no slip

Spatial distributions of the angle $\theta$ and of the displacement *u* of the belt are depicted in Figs. 5 and 6. The consideration of inertia increases the steepness of the variation of the angle and the displacement near the right touching point.

**Fig. 7:**
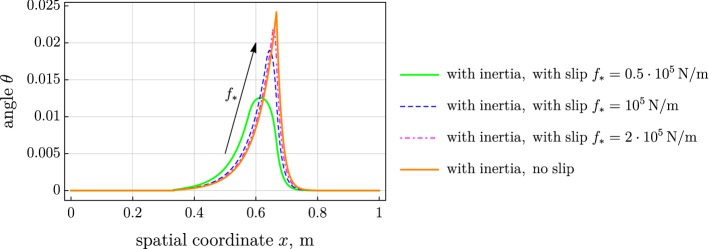
Distribution of rotation angle along the beam: convergence of slip models to stick model at increased friction force $f_*$

As the friction force in the slip region is increased, solution for the case with slip approaches the solution for the case with perfect adhesion (and the concentrated force) as expected. We illustrate this phenomenon depicting the angle $\theta$ over the spatial coordinate *x* in Fig. 7 taking three values of the prescribed friction force in the slip region: $f_* = 0.5\cdot 10^5$, $1 \cdot 10^5$, and $2 \cdot 10^5\,\mathrm {N/m}$. The remaining variables in the solutions behave similarly.

For comparison’s sake, in Table 1 we provide the computed values of the total friction force, divided into concentrated and distributed parts (or contributions from the zone of stick and the zone of slip in the third model). Here, it is seen that the inertia sufficiently reduces the overall friction force, especially its concentrated part, while the effect of slipping in general just shifts the distributed force into the “concentrated” part of the slip region.

**Table 1:** Total friction traction, *N*

	Concentrated part or slipping part in case with slip	Distributed part or sticking part in case with slip	Total
No inertia, no slip	6482	6237	12,719
With inertia, no slip	2024	5375	7398
With inertia, with slip	3253	3870	7124

## Conclusion

In the present contribution, we considered the steady state motion of a straight shear deformable beam in partial contact with a moving rough surface under the assumptions of the small strain theory. This simplified model is a prototype for the future analysis of frictional belt drive dynamics, the beam representing the belt and the rough surface being the rotating pulley. The idealized formulation and analytical (or semi-analytical) solutions allow investigating the effects of inertia and belt creep (sliding between the belt and the pulley). Transition conditions between the free segments, zone of stick contact, and zone of sliding contact play an important role in deriving the solutions.

The research presents a clear evidence for the appearance of concentrated contact interaction in the model with perfect adhesion between the belt and the moving surface of the rotating pulley. This concentrated force acts in the touching point, where the belt leaves the pulley. This has already been demonstrated earlier for the case of an axially moving string [14], but is for the first time shown for a shear deformable beam. Furthermore, we have demonstrated that the concentrated contact interaction is a limiting case of the distributed sliding friction interaction, when the sliding zone collapses into a single point because of the high friction force. Future finite element solutions for the steady state dynamic problem for a closed belt drive as well as the numerical integration of the boundary value problem similar to [4, 32] will be based on the above theoretical results and techniques of modeling.

## Footnotes

1 http://www.wolfram.com/mathematica.
